# Newborn micronutrient status biomarkers in a cluster-randomized trial of antenatal multiple micronutrient compared with iron folic acid supplementation in rural Bangladesh

**DOI:** 10.1093/ajcn/nqaa223

**Published:** 2020-08-25

**Authors:** Kerry J Schulze, Alison D Gernand, Afreen Zaman Khan, Lee S-F Wu, Sucheta Mehra, Saijuddin Shaikh, Hasmot Ali, Abu Ahmed Shamim, Pongtorn Sungpuag, Emorn Udomkesmalee, Alain B Labrique, Keith P West, Parul Christian

**Affiliations:** Center for Human Nutrition, Department of International Health, Johns Hopkins Bloomberg School of Public Health, Baltimore, MD, USA; Center for Human Nutrition, Department of International Health, Johns Hopkins Bloomberg School of Public Health, Baltimore, MD, USA; Center for Human Nutrition, Department of International Health, Johns Hopkins Bloomberg School of Public Health, Baltimore, MD, USA; Center for Human Nutrition, Department of International Health, Johns Hopkins Bloomberg School of Public Health, Baltimore, MD, USA; Center for Human Nutrition, Department of International Health, Johns Hopkins Bloomberg School of Public Health, Baltimore, MD, USA; The JiVitA Project of Johns Hopkins University, Bangladesh, Gaibandha, Bangladesh; The JiVitA Project of Johns Hopkins University, Bangladesh, Gaibandha, Bangladesh; The JiVitA Project of Johns Hopkins University, Bangladesh, Gaibandha, Bangladesh; Institute of Nutrition, Mahidol University, Bangkok, Thailand; Institute of Nutrition, Mahidol University, Bangkok, Thailand; Center for Human Nutrition, Department of International Health, Johns Hopkins Bloomberg School of Public Health, Baltimore, MD, USA; Center for Human Nutrition, Department of International Health, Johns Hopkins Bloomberg School of Public Health, Baltimore, MD, USA; Center for Human Nutrition, Department of International Health, Johns Hopkins Bloomberg School of Public Health, Baltimore, MD, USA

**Keywords:** micronutrients, pregnancy, newborn, cord blood, Bangladesh

## Abstract

**Background:**

Daily antenatal multiple micronutrient (MM) compared with iron folic acid (IFA) supplementation from early pregnancy improved birth outcomes and maternal micronutrient status in rural Bangladesh, but effects on newborn status are unknown.

**Objective:**

We examined cord blood micronutrient biomarkers in relation to antenatal MM and IFA supplementation and maternal gestational micronutrient status in rural Bangladeshi newborns.

**Design:**

In a double-blinded, cluster-randomized trial of antenatal IFA or MM (with the same IFA content), we analyzed cord blood plasma from 333 singleton births, and corresponding maternal plasma at 32.5 ± 2.6 wk of gestation, for ferritin (iron stores), folate, cobalamin (vitamin B-12), retinol (vitamin A), 25-hydroxyvitamin D [25(OH)D, vitamin D status], α-tocopherol (vitamin E), zinc, thyroglobulin, and free thyroxine (iodine status). Intervention effects and associations were determined using linear regression, exploring maternal status as a mediator of intervention effects on cord biomarkers.

**Results:**

The MM intervention increased cord ferritin (mean: +12.4%; 95% CI: 1.3, 24.6%), 25(OH)D (mean: +14.7%; 95% CI: 4.8, 25.6%), and zinc (mean: +5.8%; 95% CI: 1.0, 10.8%). Cord folate (mean: +26.8%; 95% CI: 19.6, 34.5%), cobalamin (mean: +31.3%; 95% CI: 24.6, 38.3%), 25(OH)D (mean: +26.7%; 95% CI: 23.2, 30.3%), α-tocopherol (mean: +8.7%; 95% CI: 3.6, 13.7%), zinc (mean: +2.3%; 95% CI: 0.5, 4.2%), thyroglobulin (mean: +20.1%; 95% CI: 9.0, 32.2%) and thyroxine (mean: +1.5%; 95% CI: 0.0, 3.0%) increased per 1-SD increment in maternal status (all *P* < 0.05); ferritin and retinol changed by +2.0%; 95% CI: −8.9, 14.3%; *P* = 0.72; and +3.5%; 95% CI: −0.4, 7.3%; *P* = 0.07, respectively. Ferritin, folate, cobalamin, zinc, and thyroglobulin averaged 1.57–6.75 times higher and retinol, α-tocopherol, and 25(OH)D 0.30–0.84 times lower in cord than maternal plasma, suggesting preferential maternal–fetal transfer of iron, folate, cobalamin, and zinc; limited transfer of fat-soluble vitamins; and high fetal iodine demand.

**Conclusions:**

Antenatal MM supplementation increased newborn ferritin, 25(OH)D, and zinc, while maternal and newborn folate, vitamins B-12, D, and E, zinc, and iodine biomarkers were positively related. Despite limited effects of MM, better maternal micronutrient status was associated with improved micronutrient status of Bangladeshi newborns. This trial was registered at clinicaltrials.gov as NCT00860470.

## Introduction

Micronutrients play critical roles in human reproduction, supporting gametogenesis, fertilization, embryogenesis, and placental development, function, redox balance, and vascularization (1). The essentiality and role of a given nutrient may vary across gestation—supporting increased maternal nutritional requirements or biological functions of the mother, placenta, or developing fetus, or allowing accumulation of fetal nutrient stores to sustain the newborn postnatally. The distribution of micronutrients between mother and fetus over the period of gestation must be highly regulated to ensure optimal outcomes for both, a process of coordination that could be impaired by micronutrient deficiencies. Although mechanistic functions have been attributed to micronutrients based on experimental evidence and observational studies, it has been more difficult to demonstrate linkages between specific micronutrient interventions and hypothesized maternal or infant outcomes in human trials (1). Current WHO recommendations for micronutrient supplements during pregnancy remain limited to provision of iron–folic acid (IFA) (2).

In a cluster-randomized trial in rural Bangladesh, we demonstrated that a daily antenatal multiple micronutrient (MM) supplement, containing 15 micronutrients in approximately 1-RDA amounts (3), reduced stillbirths and increased gestational age by 0.30 wk, with concomitant reductions in preterm birth and low birth weight, compared with IFA (4). These findings contributed to a meta-analysis consolidating trial data that confirmed improved pregnancy outcomes with MM relative to IFA (5). Mechanisms to explain these improved outcomes are unclear. The MM intervention in the Bangladesh trial did not improve maternal or cord blood endocrine factors related to fetal growth (6), consistent with a lack of effect of an MM supplement on cord blood endocrine factors in Burkino Faso (7) and only a modest impact of an MM intervention on placental vascular function in a trial from the Gambia (8).

In the Bangladesh trial, women in their early pregnancies (median: 10.0; IQR: 8.1, 12.9 wk) were anemic (20%), with deficiencies of iron (4.0%); folate (2.5%); vitamins A (6.7%), B-12 (35%), D (64% insufficiency), and E (58%); zinc (13%); and iodine (2.6%) (9). There was a benefit of the MM compared with IFA intervention on maternal micronutrient status, although deficiencies were not eliminated, in late pregnancy (9). In this study, our primary aim was to examine whether the antenatal MM compared with IFA supplementation affects newborn micronutrient biomarker concentrations in cord blood. We also explored relations between cord blood and maternal late-pregnancy micronutrient status biomarkers, as a proxy for maternal to fetal micronutrient transfer. An impact of MM compared with IFA could reflect the preferential transfer of a nutrient to the fetus regardless of its impact on maternal status, or could be mediated by improvements in maternal status for nutrients that were particularly responsive to supplementation. These questions have not been explored comprehensively in trials of antenatal MM compared with IFA supplementation, and findings will demonstrate the potential benefits of maternal antenatal micronutrient supplementation on newborns in a setting where micronutrient deficiencies are common.

## Methods

### Population and design

This study is derived from a double-blinded, cluster-randomized, controlled trial, called JiVitA-3, conducted at a population research site in the Gaibandha District in northwest Bangladesh, as previously described (4). The GIS-mapped study area was divided into 596 sectors, which served as the unit of randomization. Married women, if found to be pregnant by urine testing for human chorionic gonadotrophin hormone during 5 weekly pregnancy surveillance rounds, consented to participate and were provided with supplements according to their sector randomization. Supplements were either the IFA (standard of care, containing 27 mg iron and 600 µg folic acid) or MM, which contained approximately 1-RDA amounts of vitamins A (770 µg retinol activity equivalents), D (5 µg), E (15 mg), B1 (thiamin, 1.4 mg), B2 (riboflavin, 1.4 mg), B3 (niacin, 18 mg), B6 (1.9 mg), B12 (2.6 µg), C (85 mg), and the minerals zinc (12 mg), copper (1 mg), selenium (60 µg), and iodine (220 µg), as recommended (3), as well as the same amounts of iron and folic acid (27 mg and 600 µg, respectively). The premix for these supplements was produced gratis by DSM, and tablets were produced, labeled, and packed in opaque bottles gratis by Beximco Pharmaceuticals. At the field headquarters, supplements were stored under controlled temperature and humidity until use. Quality and content of the nutrients in the supplements were checked 2 to 3 times annually by an independent laboratory. Coded supplements were replenished on a weekly basis for daily self-administration and compliance was high (4).

### Trial substudy cord blood collection

Within the entire study area of 596 sectors, 64 contiguous and centrally located sectors were selected for more extensive assessment of participants, including biospecimen collection to assess maternal vitamin and mineral status. Maternal venous blood was collected at the time of enrollment (presupplementation), typically 10 wk of gestation, and at 32 wk of gestation, as described (9). Within this substudy area, 31 sectors were selected from which women were additionally enrolled into a protocol to include home-based cord blood collection, as described (6).

The protocol for venous cord blood collection and processing has also been described (6). In this rural setting, women typically delivered their infants at home, necessitating a labor tracking system through which study teams would be notified when a woman began labor. Trained study midwives arrived in the home (or a facility for a few participants), assisted with deliveries, and collected cord venous blood (7 mL) as soon as birth was completed and separation of the infant and placenta was done. Blood was collected via a syringe and transferred to a blood collection tube; put in a padded, insulated cold box; and transported to the field-based laboratory for processing, typically 1–2 hours after collection. Both maternal and cord blood were centrifuged and plasma separated, and multiple aliquots were stored in liquid nitrogen before transit to Johns Hopkins University in the United States or the Institute of Nutrition at Mahidol University in Thailand for analysis. Trace element free supplies were used and samples were protected from light exposure throughout sample collection and processing.

A Consolidated Standards of Reporting Trials diagram was published (6) and is updated in **Supplementary Figure 1**, wherein 333 samples from singleton births were available from 500 enrolled women, with few refusals and comparable loss to follow-up by intervention. The a priori goal was 155 per intervention group to test for detectable differences of 0.35 SD in micronutrient biomarkers with α = 0.05 and power = 0.80, assuming a design effect (due to cluster randomization) of 1.20, based on prior experience. Participants in the cord blood collection substudy were similar to those who participated in the larger substudy, and comparisons of both the cord blood study (6) and the larger substudy participants (9) to the main JiVitA-3 trial cohort (4) have been described.

All procedures were approved by the Institutional Review Board at Johns Hopkins Bloomberg School of Public Health in Baltimore, MD, USA, and the Bangladesh Medical Research Council in Dhaka, and consent was obtained from each participant. The JiVitA-3 trial enrolled participants beginning in January 2008 (4), the main substudy sought consent for enrolled participants from June 2008 to May 2010 (9), and the cord blood collection activity sought consent from a subset of those participants from February 2009 to April 2010, with the last contributing births occurring in November 2010 (6).

### Laboratory assessments of outcomes

Analysis of maternal samples collected at 32 wk of gestation has been described (9). For cord blood plasma, all samples were analyzed at Johns Hopkins Bloomberg School of Public Health using ultra-HPLC (Acquity H Class, Waters Corporation) for retinol and α-tocopherol using an adaptation of common methods (10) and validated using SRM 968d [National Institutes of Standards and Technology (NIST)]. Ferritin (reflecting iron stores), folate, and total cobalamin (vitamin B-12) were analyzed by chemiluminescent immunoassay (Immulite 2000, Siemens Diagnostics). Thyroglobulin (elevated when demand for iodine is high) and free thyroxine (fT4; a thyroid hormone; maternal fT4 is required by the fetus in early pregnancy) as biomarkers of iodine status were also analyzed by chemiluminescent immunoassay (Immulite 2000, Siemens Diagnostics). 25-Hydroxyvitamin D [25(OH)D] was assessed by commercial immunoassay (IDS, Inc.). For plasma zinc, graphite furnace atomic absorption spectroscopy was used (AAnalyst 800, Perkin Elmer), with an assay validated against SRM 1598 (NIST) and run with standards produced from a lyophilized human serum product (Seronorm, SERO). As a marker of inflammation, α1-acid glycoprotein (AGP) was analyzed with a commercial radial immunodiffusion assay (Kent Laboratories). All assays were run with commercially prepared or in-house quality control materials, and typical intra- and interassay CVs have previously been reported (11). Incomplete data for cord blood biomarkers are due to insufficient plasma, cases for which corresponding maternal values were unavailable, or out of range values. For plasma zinc, this included samples with measured values >30.0 µmol/L (1 in cord blood; 3 in maternal plasma at 32 wk of gestation), and for fT4 included samples with values >32.0 pmol/L (1 in maternal plasma). Samples were stored at −80°C in multiple aliquots, and all assays were completed within 5 years of receipt with aliquot use organized to optimize sample integrity.

To our knowledge, conventional validated cutoffs have not been established for describing newborn micronutrient deficiencies in cord blood plasma. However, where cutoffs have been used in the literature [e.g., ferritin <34 µg/L (12), retinol <0.35 or <0.70 µmol/L, and α-tocopherol <7.5 µmol/L (13); 25(OH)D <30 or 50 nmol/L (14, 15)] we report the prevalence affected. We also describe distributions of all cord blood plasma biomarkers.

### Data analysis

Maternal and newborn characteristics that may have had bearing on newborn biomarkers are presented as mean ± SD or percentage and were examined by intervention to establish comparability between interventions using linear or logistic regression.

Data on micronutrient status biomarkers are presented as mean ± SD or, to best capture the central tendencies and asymmetry of skewed distributions, geometric mean (−1 SD, +1 SD). Quantile normal plots, measures of skewness and kurtosis, and improved homoscedasticity of residual plots in regression analysis justified log_10_ transformations of cord blood and corresponding maternal biomarkers for analytes other than retinol, α-tocopherol, and thyroxine. Table footnotes are used to demonstrate calculations, particularly for log_10_-transformed variables. The effect of maternal antenatal MM supplementation on cord blood biomarkers was tested with a linear regression model that included intervention status (0 = IFA, 1 = MM). The intervention effect is presented both as the mean difference (95% CI) in biomarker concentrations in their units of measurement and as the percentage (95% CI) difference in the MM relative to the IFA group to compare the magnitude of impact across biomarkers. For retinol, α-tocopherol, and thyroxine the concentration difference is the β-coefficient (95% CI) associated with the intervention. For log_10_-transformed biomarkers, the concentration difference was calculated by back-transforming regression parameters and 95% CIs to the arithmetic scale. The percentage difference associated with MM for variables on the arithmetic scale was calculated as 100 × β_1_(95% CI)/β_0_, since β_1_ is the difference from IFA (β_0_) associated with MM. Finally, percentage difference for log_10_-transformed variables took advantage of the multiplicative nature of the logarithmic scale (16), whereby 10^β1(95% CI)^ could be utilized to calculate the fold difference in biomarkers associated with MM compared with IFA, then expressed as a percentage (95% CI). Finally, logistic regression was used to test the prevalence of newborn deficiency by intervention group when those outcomes are reported.

Associations of cord with maternal micronutrient biomarkers at 32 wk of gestation are depicted with scatterplots and best-fit lines by intervention group. In exploratory analyses, associations between maternal and cord biomarkers, as outcomes, were tested with linear regression. Distributions of maternal biomarkers were standardized to mean±SD = 0 ± 1 to compute the percentage difference in cord blood per 1-SD increment in the maternal biomarker. For log_10_-tranformed biomarkers, the fold change in biomarker per any 1-SD increment in maternal status is calculated by 10^β1(95% CI)^, where β_1_ is the change in cord blood biomarker per increment in maternal biomarker, expressed as a percentage. For retinol, α-tocopherol, and thyroxine, for which associations are not on a multiplicative scale, the percentage increase associated with a change in maternal status was calculated from 0 (the mean) to +1-SD of the maternal biomarker distribution as 100 x β_1_(95% CI)/β_0_, with β_1_ the change in the cord blood per 1-SD gain in maternal biomarker and β_0_ the value for the cord blood indicator at the mean of the maternal distribution.

For biomarkers for which both intervention and maternal status effects were observed [25(OH)D and zinc], models that included both as determinants of newborn status were examined using the “medeff” command to ascertain extent to which the intervention effect was mediated through improved maternal micronutrient status (17–19).

Models including variables that could influence newborn micronutrient biomarkers (maternal early pregnancy micronutrient status, exact gestational age of late pregnancy maternal blood draw, late pregnancy and cord blood AGP, gestational age and weight, length, and weight-for-length *z*-score at birth) were also examined. They did not change fundamental findings of maternal–newborn associations and are only shown as supplementary output when the additional variables were statistically significant. No adjustments were made to maternal biomarkers (e.g., ferritin or retinol) based on AGP concentrations to account for inflammation because associations between AGP and all micronutrient biomarkers were lacking at 32 wk of gestation.

Finally, we calculated the ratio of the cord blood biomarker to the maternal biomarker at 32 wk of gestation, and present the distribution of values as mean ± SD or geometric mean (−1-SD, +1-SD), to serve as a proxy for the transfer of nutrients from mother to fetus in late gestation.

Analyses were conducted using Stata 13.0 (StataCorp), and *P* < 0.05 was considered statistically significant. Robust SEs to account for cluster randomization were calculated in all regression models.

## Results

Data on maternal characteristics at the onset of pregnancy and newborn status of the 333 participants are presented in **Table 1**. In this rural region, mothers were young and undernourished, with short stature and low BMI. There was no difference by intervention group in maternal or newborn characteristics other than birth length, which was greater among those in the IFA than those in the MM group. Gestational age at birth did not differ by intervention, and length was greater in the IFA group, unlike findings from the main trial, for which both were greater in the MM group (4). Maternal biomarker distributions for this subcohort are shown by intervention group in **Supplementary Table 1**, demonstrating a benefit of the MM intervention on maternal retinol, 25(OH)D, α-tocopherol, and zinc, albeit with largely overlapping distributions, at 32 wk of gestation.

**TABLE 1:** Characteristics of mothers and newborns by intervention in the cord blood substudy of the JiVitA-3 trial in rural Bangladesh^1^

Characteristic	All (*n* = 333)	IFA (*n* = 157)	MM (*n* = 176)	*P* value
Maternal				
Age, y	23.5 ± 5.3	23.8 ± 5.5	23.2 ± 5.1	0.43
Weight, kg	43.4 ± 6.5	44.1 ± 7.4	42.7 ± 5.6	0.12
Height, cm	148.9 ± 5.3	149.2 ± 5.5	148.7 ± 5.1	0.56
BMI,^2^ kg/m^2^	19.5 ± 2.5	19.8 ± 2.5	19.3 ± 2.2	0.12
Gestational age at 1st assessment, wk	10.6 ± 3.8	10.8 ± 4.2	10.4 ± 3.4	0.50
Gestational age at late pregnancy assessment, wk	32.5 ± 2.6	32.7 ± 2.3	32.3 ± 2.8	0.16
Newborn				
Sex, male	54.0	53.5	54.4	0.93
Gestational age at birth, wk	39.3 ± 2.8	39.4 ± 2.4	39.1 ± 3.1	0.40
Birth weight, kg	2.66 ± 0.41	2.70 ± 0.43	2.62 ± 0.39	0.10
Birth length, cm	47.3 ± 2.1	47.6 ± 2.2	47.0 ± 2.1	0.02
Weight-for-age *z* score	−1.48 ± 0.96	−1.40 ± 0.99	−1.57 ± 0.94	0.13
Length-for-age *z* score	−1.38 ± 1.10	−1.19 ± 1.12	−1.47 ± 1.06	0.02
Weight-for-length *z* score	−0.84 ± 0.96	−0.89 ± 0.94	−0.79 ± 0.98	0.30

1 Values are mean ± SD or, for sex, percentage, with comparisons between the IFA and MM groups assessed by regression, with intervention as the independent variable and with robust SEs to account for cluster randomization; missing observations: *n* = 2 for gestational age and first and late pregnancy assessments; *n* = 8 for gestational age at birth; *n* = 2 for birth weight; *n* = 5 for birth length; *n* = 41 for weight-for-length *z* score. IFA, iron folic acid; MM, multiple micronutrient.

2 BMI reported in early pregnancy (enrollment), in the absence of prepregnancy BMI. IFA, iron folic acid; MM, multiple micronutrients.

Cord plasma biomarker concentrations are shown in **Table 2** by antenatal intervention group, along with the difference in values between the MM and IFA groups. Despite the same amounts of iron and folate in the IFA and MM supplements, cord blood ferritin was 18 µg/L, or 12.4% (*P* = 0.03), higher in the MM than the IFA newborns, and mean folate was 5 nmol/L, or 7.5%, lower, although not significantly (*P* = 0.07). Among other nutrients, only 25(OH)D (14.7%, *P* = 0.004) and zinc (5.8%, *P* = 0.02) were increased in cord blood by the MM intervention. Differences in vitamin B-12, retinol, vitamin E, and iodine status indicators by intervention group were all associated with *P* > 0.05, although point estimates for vitamins B-12 and E in particular were positive. AGP did not differ by intervention [geometric mean (−1-SD, +1-SD): 0.20 (0.12, 0.33) compared with 0.22 (0.14, 0.34) g/L for IFA and MM, respectively, *P* = 0.156].

**TABLE 2:** Newborn (cord blood plasma) micronutrient biomarker concentrations and their differences by maternal antenatal intervention in the JiVitA-3 trial in rural Bangladesh^1^

	IFA		MM		Difference between MM and IFA			
Nutrient	*n*	Mean ± SD or geometric mean (−1 SD, +1 SD)	*n*	Mean ± SD or geometric mean (-1 SD, +1 SD)	Biomarker units, 95% CI	%, 95% CI	*P* value	*R^2^*
Iron								
Ferritin, µg/L	153	142 (67, 301)	167	160 (85, 299)	+18 (2, 35)	+12.4 (1.3, 24.6)	0.03	0.0071
Folate								
Total plasma folate, nmol/L	156	61 (39, 96)	176	56 (35, 90)	−5 (−9, 0)	−7.5 (−14.9, 0.6)	0.07	0.0072
Vitamin B-12								
Cobalamin, pmol/L	155	286 (168, 489)	176	309 (182, 527)	+23 (−10, 60)	+8.0 (−3.5, 21.0)	0.17	0.0052
Vitamin A								
Retinol, µmol/L	134	0.63 ± 0.20	148	0.65 ± 0.25	+0.03 (−0.04, 0.09)	+4.1 (−6.3, 14.4)	0.43	0.0032
Vitamin D								
25(OH)D, nmol/L	157	38 (27, 54)	176	44 (31, 62)	+6 (2, 10)	+14.7 (4.8, 25.6)	0.004	0.0381
Vitamin E								
α-Tocopherol, µmol/L	134	5.7 ± 2.0	148	6.2 ± 2.0	+0.4 (−0.2, 1.1)	+7.7 (−3.3, 18.8)	0.16	0.0119
Zinc								
Total plasma zinc, µmol/L	155	14.3 (11.7, 17.5)	176	15.2 (12.6, 18.4)	+0.8 (0.1, 1.6)	+5.8 (1.0, 10.8)	0.02	0.0214
Iodine								
Thyroglobulin, µg/L	149	35.3 (16.2, 77.2)	170	35.2 (18.6, 66.5)	−0.2 (−5.2, 5.8)	−0.4 (−14.7, 16.3)	0.96	0.0000
fT4, pmol/L	149	15.3 ± 1.7	171	15.2 ± 1.6	−0.2 (−0.6, 0.2)	−1.1 (−3.9, 1.6)	0.40	0.0028

1 Geometric mean (−1 SD, +1 SD) was calculated by back-transforming from the log_10_ scale: mean ± SD for log_10_-transformed ferritin in the IFA group is 2.153 ± 0.325, so 10^2.153^ (10^2.153–0.325^, 10^2.153+0.325^) = 142 (67, 301). Differences in biomarker values between MM and IFA groups were assessed by linear regression with intervention (0 = IFA, 1 = MM) as the independent variable with robust SEs to account for cluster randomization. For biomarkers on the arithmetic scale the MM effect = β_1_ (95% CI) from the regression model. For log_10_-transformed variables, the MM effect = 10^β0+β1^–10^β0^, where β_0_ is the regression constant for the IFA group and β_1_ the MM effect or the upper or lower bound of its 95% CI: for ferritin, β_0_ = 2.1528, β_1_ (95% CI) = 0.0506 (0.0057, 0.0955), so 10^2.1528+0.0506^–10^0.0506^ = 18, then substituting upper or lower bounds of 95% CI for β_1_. On the arithmetic scale, 100 × β_1_(95% CI)/β_0_ was used to calculate percentage difference in values between the MM and IFA groups: for retinol, β_0_ = 0.6289, β_1_ (95% CI) = 0.0257 (−0.0394, 0.0908), so 100 × 0.0257/0.6289 = 4.1%, then substituting upper and lower bounds of 95% CI for β_1_. For log_10_-transformed biomarkers, percentage change = 10 ^β1(95% CI)^: for ferritin, 10^0.0506^ (10^0.0057^, 10^0.0955^) = 1.124 (1.013, 1.246), indicating a 12.4 (95% CI: 1.3, 24.6)% increase in ferritin in the MM compared with IFA group after subtracting 1 and multiplying by 100 (16).

Conversion of biomarker units can be done as follows: folate nmol/L × 0.4413 to ng/mL, cobalamin pmol/L × 1.355 to pg/mL, retinol µmol/L × 28.65 to µg/dL, 25(OH)D pmol/L × 0.4006 to ng/mL, α-tocopherol µmol/L × 0.423 to µg/mL, zinc µmol/L × 6.534 to µg/dL, fT4 pmol/L × 0.0777 to ng/dL. fT4, free thyroxine; IFA, iron folic acid; MM, multiple micronutrients; 25(OH)D, 25-hydroxyvitamin D.

Using published cutoffs, 4.6% of IFA and 1.8% of MM newborns had ferritin <34 µg/L (*P* = 0.10 for difference by logistic regression), 67.9% and 66.2%, respectively, had retinol <0.70 µmol/L (*P* = 0.76), with 5.2% and 10.6% <0.35 µmol/L (*P* = 0.11); 82.8% and 75.5% had cord α-tocopherol < 7.5 µmol/L (*P* = 0.29). For 25(OH)D, 77.1% of newborn values in the IFA group and 64.2% in the MM group (*P* = 0.010) were <50 nmol/L, and 24.8% compared with 10.8% (*P* = 0.001) were <30 nmol/L, respectively, by logistic regression. Percentiles for each micronutrient biomarker are presented in **Supplementary Table 2**.

**FIGURE 1:**
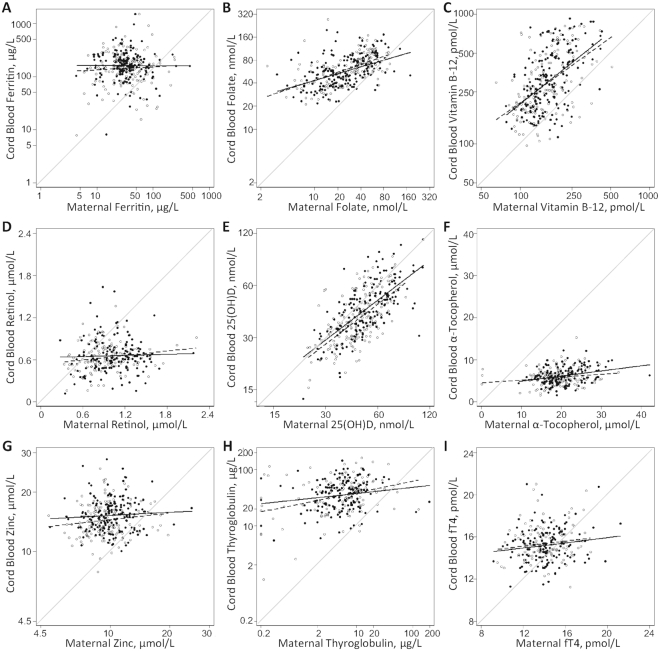
Associations of cord blood micronutrient status biomarkers with maternal micronutrient biomarkers assessed at 32 wk of gestation among mother–newborn pairs in rural Bangladesh in the context of a trial of antenatal IFA and MM supplementation. Micronutrient status biomarkers include ferritin (A), folate (B), vitamin B-12 (C), retinol (D), 25(OH)D (E), α-tocopherol (F), zinc (G), thyroglobulin (H), and free thyroxine (I). Scatterplots and best fit lines are shown by intervention group such that open circles and dashed lines denote the IFA group and solid lines and closed circles denote the MM group. The gray line that diagonally crosses each image represents where maternal and newborn biomarker concentrations are the same. IFA, iron folic acid; MM, multiple micronutrients; 25(OH)D, 25-hydroxyvitamin D.

**Figure 1** shows associations of newborn status with maternal status for each biomarker by intervention group, which largely overlap. All fitted lines show mean newborn values at or above the unity line at the lowest concentrations of maternal status, but varying slopes in relation to maternal status. The strength of association between maternal and newborn status, excluding intervention, is quantified in **Table 3**. Only for ferritin was evidence of an association between maternal and newborn status absent, with a positive but NS association of maternal–newborn retinol. Maternal–newborn associations of folate, vitamin B-12, 25(OH)D, and thyroglobulin were strongest (all *>*20% difference per 1-SD increment in maternal biomarker) and those for free thyroxine were weakest (<2% difference). Maternal status explained ∼45% of the variability in cord blood 25(OH)D and ∼26% of the variability in folate and vitamin B-12 (as *R*^2^). There was an intermediary association of maternal status with α-tocopherol and thyroglobulin, both explaining >6% of the variability in newborn values, despite the ∼20% change in thyroglobulin but only an 8.7% change in α-tocopherol per 1-SD shift in maternal status. The association of maternal zinc with cord zinc concentrations was small yet significant (*P* = 0.015), with an ∼2% increase in cord blood zinc concentration per 1-SD shift in maternal status.

**TABLE 3:** Concentrations of maternal and newborn (cord blood plasma) micronutrient status biomarkers, their strength of association, and the ratio of newborn to maternal biomarker concentrations assessed at 32 wk gestation among participants in the JiVitA-3 trial in rural Bangladesh^1^

Biomarker	*n*	Maternal, 32-wk gestation	Cord blood	Cord blood biomarker difference per 1-SD maternal biomarker increment, % (95% CI)	*P* value	*R* ^2^	Cord:maternal ratio, % (95% CI)
Ferritin, µg/L	317	32 (16, 66)	151 (76, 302)	+2.0 (−8.9, 14.3)	0.72	0.0008	4.66 (1.74, 12.45)
Folate, nmol/L	329	26 (12, 54)	59 (37, 93)	+26.8 (19.6, 34.5)	<0.0001	0.2657	2.28 (1.20, 4.35)
Cobalamin, pmol/L	328	163 (113, 233)	297 (175, 506)	+31.3 (24.6, 38.3)	<0.0001	0.2625	1.83 (1.15, 2.91)
Retinol, µmol/L	279	0.99 ± 0.32	0.64 ± 0.23	+3.5 (−0.4, 7.3)	0.073	0.0100	0.64 (0.40, 1.03)
25(OH)D, nmol/L	330	49 (36, 66)	41 (29, 59)	+26.7 (23.2, 30.3)	<0.0001	0.4501	0.84 (0.64, 1.10)
α-Tocopherol, µmol/L	279	19.9 ± 5.1	6.0 ± 2.0	+8.7 (3.6, 13.7)	0.0014	0.0626	0.30 (0.17, 0.54)
Zinc, µmol/L	326	9.9 (7.8, 12.4)	14.9 (12.2, 18.0)	+2.3 (0.5, 4.2)	0.015	0.0141	1.57 ± 0.45
Thyroglobulin, µg/L	279	5.1 (1.6, 15.8)	34.4 (17.0, 69.8)	+20.1 (9.0, 32.2)	0.0005	0.0671	6.75 (2.10, 21.66)
fT4, pmol/L	261	14.1 ± 1.9	15.3 ± 1.7	+1.5 (0.0, 3.0)	0.044	0.0200	1.09 ± 0.18

1 Values for maternal and cord blood biomarkers are averaged across intervention groups and expressed as geometric means (−1-SD, +1-SD), as described for Table 2, or mean ± SD. Percentage difference in cord blood micronutrient biomarkers is expressed per 1-SD increment in maternal values with simple linear regression with maternal biomarkers as independent variables standardized to mean ± SD = 0 ± 1. For biomarkers on the arithmetic scale, percentage difference is calculated from 100 × β_1_(95% CI)/β_0_, where β_0_ is the cord biomarker value at the mean of the maternal distribution and β_1_ the change per 1-SD maternal biomarker; calculations are 100 × 0.0224 (−0.0023, 0.0471)/0.6442 µmol/L for retinol, 100 × 0.5164 (0.2175, 0.8153)/5.9667 µmol/L for α-tocopherol, and 100 × 0.2324(0.0062, 0.4585)/15.2726 pmol/L for fT4. Percent difference for log_10_-transformed variables is 10^β1 (95% CI)^: for ferritin, β_1_ (95% CI) of 0.0087(−0.0407, 0.0582) for a 1-SD increment in maternal ferritin results in a 10^0.0087^(10^−0.0407^, 10^0.0582^) = 1.020 (0.911, 1.143)-fold change in cord ferritin, or a 2.0 (−8.9, 14.3)% increase. The ratio of cord to maternal biomarker concentrations is expressed as geometric means (−1-SD, +1-SD) or mean ± SD, depending on whether log_10_-transformation was required to normalize the distribution or not, respectively. fT4, free thyroxine; IFA, iron folic acid; MM, multiple micronutrients; 25(OH)D, 25-hydroxyvitamin D.

In mediation models (**Supplementary Table 3**), both the MM intervention [β (95% CI): 0.022 (0.002, 0.042); *P* = 0.035] and maternal zinc concentrations [β (95% CI): 0.008 (0.000, 0.016); *P* = 0.045] remained independent determinants of cord blood zinc, with 11.3% (95% CI: 6.2, 50.5) of the total effect on newborn status explained by mediation through maternal status. For 25(OH)D, only maternal status retained significance as a determinant of newborn 25(OH)D [0.101 (0.088, 0.113); *P* < 0.0001], with an estimated 62.4% (95% CI: 38.6, 180.0) of the intervention effect mediated through improved maternal status.

Additional findings (**Supplementary Table 4**) include a positive association of newborn retinol with size at birth, a positive association of AGP with retinol and α-tocopherol in cord blood, and a stronger effect of early than late pregnancy thyroxine on newborn thyroxine. Maternal and fetal AGP were not associated with each other [β (95% CI): −0.024 (−0.222, 0.175); *P* = 0.810 for regression between log_10_-transformed values].

Table 3 also shows the ratio of cord blood to maternal nutrient biomarkers across intervention groups combined. Ferritin averaged nearly 5 times higher in cord than maternal plasma, suggesting accumulation of iron stores in the fetus relative to the mother. Folate, cobalamin, zinc, and, less so, thyroxine, were higher on average in cord than maternal plasma. Thyroglobulin was nearly 7 times higher in cord blood than maternal circulation. Conversely, the fat soluble vitamins averaged 16% (for vitamin D) to 70% (for vitamin E) lower in cord blood than maternal circulation at 32 wk gestation. Cord blood AGP was 0.37 (95% CI: 0.20, 0.69) times, i.e., 63% lower than, maternal values. For all biomarkers, concentrations were significantly different between mothers and newborns by paired *t*-test.

## Discussion

This study revealed newborn micronutrient status in a typical rural South Asian setting in a trial of antenatal MM compared with IFA and in relation to maternal status. We found that a daily supplement providing an RDA of 15 micronutrients improved newborn status of ferritin, zinc, and vitamin D compared with IFA, but not the other micronutrients we examined. The MM intervention enhanced newborn iron and zinc status directly, and vitamin D through improved maternal status. Maternal micronutrient status was typically the major determinant of newborn status, with maternal–newborn associations of folate, vitamins B-12, D, and E (but less so retinol), and zinc and iodine status. Adaptive mechanisms seemed to ensure a modicum of fetal nutriture among most poorly nourished mothers. Findings are further explored for each nutrient.

An unexpected impact of the MM intervention was 12% higher cord ferritin despite the same iron content of the IFA and MM supplements. Along with modestly reduced late pregnancy ferritin observed in a larger group of MM mothers (9), this suggests a subtly enhanced efficiency of iron transfer to the fetus in the presence of micronutrients beyond iron and folic acid. While requiring cautious interpretation and confirmation, this mechanism could be associated with longer gestation (4) or greater birth size (5) observed in MM supplementation trials. Eighty percent of fetal iron stores accumulate in the last trimester (20), and ferritin nearly 5 times higher in fetal than maternal circulation is consistent with prioritization of iron to the fetus when poor maternal status as a limiting factor is absent (20, 21). Iron deficiency is uncommon in this community in Bangladesh (22, 23). Lack of a maternal–newborn ferritin association is consistent with data from iron-replete women (24, 25), and cord ferritin was comparable to that of healthy term infants (26, 27).

Maternal and newborn folate were strongly associated, with cord folate double that of folate in mothers. Cord folate of Bangladeshi infants was similar to that observed in folic acid supplementation studies, which also showed that cord folate predominantly comprises the reduced, active metabolites rather than unmetabolized folic acid (28, 29), a distinction we could not make. Accumulation of 5-methyl tetrahydrofolate at the placental surface allows folate transfer down a concentration gradient to the fetus (30, 31) via folate receptor-α, a folate transporter, and reduced folate carrier (32, 33). Expression of receptors increases to enhance transfer over gestation (33).

Maternal and cord blood cobalamin were also strongly associated, with approximately double the concentration in cord blood, as previously observed (34–38) and reviewed (39). Relations are consistent with active transport of the vitamin, which, like folate, accumulates at the placental surface (40), with increased mRNA expression of a placental transcobalamin associated with greater cord blood vitamin B-12 (41). Reported mean cord blood vitamin B-12 ranges from ∼100 (37) to 600 (41) pmol/L. In this study values were in the middle of that range, despite a >30% prevalence of maternal B-12 deficiency at entry into pregnancy and declines in maternal B-12 even with benefits of the MM supplement (9). Our findings support vitamin B-12 prioritization for the fetus, but with newborn status strongly contingent on maternal concentrations.

Unlike the B-vitamins, fat-soluble vitamins were lower in newborn than maternal circulation, as previously observed (42). Cord retinol averaged ∼64% of maternal values, the MM impact on cord concentrations was negligible despite its effect on maternal status (9), and the association of cord with maternal retinol was weak. Our findings are generally consistent with observations in vitamin A deficient Brazilian (43) and sufficient South African (44) mother–newborn dyads. Vitamin A may be distributed to the placenta as retinol or retinyl esters, utilized as retinoids or stored as retinyl esters, and distributed to fetal tissue via cellular retinol binding proteins I and II for tissue differentiation and organogenesis in early pregnancy or later for fetal uptake (45–47). The various forms and pathways may preclude a tight correlation between maternal and newborn status, and could explain the observed association of infant size with newborn retinol. Infants are known to be born with limited vitamin A stores, and the status of the Bangladeshi neonates was similar to that of healthy European infants (48). Rather, breast milk is quantitatively a more significant source of vitamin A for the infant (49).

Similarly, cord α-tocopherol was only 30% of that in maternal circulation but was associated with maternal status, in agreement with known aspects of maternal–fetal vitamin E metabolism (45, 50). Transfer of maternal to cord α-tocopherol is mediated by the placental α-tocopherol transfer protein (51–53) and may be limited by several factors. Maternal α-tocopherol is associated with circulating lipids and thereby becomes elevated during gestation (9), but lipids are not efficiently transferred across the placenta (50). Also, there is high specificity for transfer of the naturally occurring, or RRR, stereoisomer of α-tocopherol, while supplements typically include other stereoisomers (54). Finally, α-tocopherol distributed throughout fetal tissues and red blood cells may be more tightly linked with maternal status (50); thus, cord blood cutoffs may not reflect actual status. Here, mean cord blood α-tocopherol was similar to that of European infants (48). As with retinol, colostrum and breast milk are important sources of vitamin E in infancy (45, 50).

Cord 25(OH)D was related to the MM intervention, strongly associated with maternal concentrations, and ∼80% of maternal values, which are responsive to supplementation (9, 55). Similar associations have been observed previously (14, 15, 56). Vitamin D has roles in placental immunomodulation and cellular differentiation (57, 58) and calcemic effects on fetal bone development (57), and the placenta is a major site for 25(OH)D conversion to 1,25(OH)2D (57). Thus, some placental utilization of 25(OH)D may explain its lower concentrations in cord than maternal blood. Optimizing transfer of 25(OH)D to the fetus in utero is critical since the amounts of vitamin D in colostrum and breast milk are low (59). Vitamin D deficiency is prevalent regionally (9, 56), arguing for higher supplemental intakes (15, 56). Even with the MM intervention, 11% of newborns were vitamin D deficient.

The MM intervention and maternal status were independently associated with newborn zinc status, with higher zinc in cord blood than maternal circulation. With progression of pregnancy, affinity of serum albumin for zinc declines (60), possibly allowing more free zinc for placental uptake, even as maternal zinc is redistributed to maternal blood cells (60). The mechanism of placental transfer is likely to occur via a zinc importer protein family (SLC39) and zinc exporter protein family (SLC30), which are responsive to maternal status to ensure zinc adequacy of the fetus (21, 60). Cord blood zinc averages ∼14 µmol/L, typically higher than maternal circulating zinc (60), although it was recently reported as ∼20 µmol/L in European neonates (48). That the MM supplement improved newborn zinc, which was similar to reported values despite maternal deficiency, is consistent with preferential and highly regulated transfer to the fetus.

Newborn thyroxine and thyroglobulin did not differ by intervention, and both were positively associated with maternal concentrations. Thyroxine from the mother, which is transferred in proportion to maternal production, is required for fetal brain development early in pregnancy (61). Despite declining contributions of maternal thyroxine over gestation, fetal fT4 at delivery is of both maternal and fetal origin (61), potentially explaining the stronger association of cord fT4 with early pregnancy maternal concentrations. High newborn relative to maternal thyroglobulin is typical and may reflect iodine demand (62). Others have observed impacts of antenatal iodine supplementation on cord blood thyroglobulin (63–65), suggesting that more iodine in the MM supplement could be warranted.

Although determining intervention effects was our primary aim, biomarker associations from exploratory analyses enhance understanding of micronutrient pathways in the maternal–newborn dyad; unmeasured biological mechanisms are likely at play where the contribution of intervention or maternal status to characterizing cord biomarkers is small. We were not able to measure biomarkers for all of the micronutrients in the MM supplement or all metabolites of interest, and there is a limited ability to characterize infant status with cord blood micronutrient biomarkers, including AGP. However, our findings provide a basis for interpreting newborn data from similar contexts in future studies. Finally, we cannot eliminate the possibility that participants represented a biased sample given that they were derived from a limited geographic location and time period within the larger randomized trial, although intervention groups were comparable across a variety of factors.

Integration of findings from the JiVitA-3 trial shows that an antenatal MM supplement confers important public health benefits in rural Bangladesh, including reduced preterm birth and low birth weight through longer gestation (4) rather than growth-enhancing endocrine pathways (6). MM supplementation at ∼1 RDA reduces, but does not eliminate, maternal micronutrient deficiencies (9) and, based on the present analysis, exerts modest direct effects on newborn micronutrient status, with higher ferritin in newborns of MM supplemented mothers a novel finding. Findings from the JiVitA-3 trial to date support the efficacy of an antenatal MM supplement in improving pregnancy outcomes, while raising a question about potentially even greater efficacy of providing amounts exceeding current RDAs to normalize the micronutrient status of the maternal–newborn dyad in undernourished populations, for which pregnancy-specific studies are lacking and greatly needed.

## Supplementary Material

nqaa223_Supplemental_File

## Notes

Funded by the Bill & Melinda Gates Foundation OPP614 (Global Control of Micronutrient Deficiency) and OPP1141435 (Improving Adolescent Newlywed Nutrition and Health for Pregnancy in Bangladesh and Health across Early Life Stages in South Asia). Additional assistance was received from the Sight and Life Global Nutrition Research Institute, Baltimore, MD, USA; DSM NV, Kaiseraugst, Switzerland, Bombay, India, and Singapore provided in-kind support by formulating and delivering micronutrient premixes for supplement production and testing for potency free of charge. Beximco Pharmaceuticals, Ltd., Dhaka, produced, bottled, labeled and delivered micronutrient and placebo supplement tablets to the field site free of charge.

Data described in the manuscript, code book, and analytic code will be made available upon request pending application and approval.

Supplementary Tables 1–4 and Supplementary Figure 1 are available from the “Supplementary data” link in the online posting of the article and from the same link in the online table of contents at https://academic.oup.com/ajcn/.

Abbreviations used: AGP, α1-acid glycoprotein; fT4, free thyroxine; IFA, iron folic acid; MM, multiple micronutrients; NIST, National Institute of Standards and Technology; SRM, Standard Reference Material; 25(OH)D, 25-hydroxyvitamin D.
