# Supplementary material for: Newborn micronutrient status biomarkers in a cluster-randomized trial of antenatal multiple micronutrient compared with iron folic acid supplementation in rural Bangladesh
Source: Am J Clin Nutr. 2020 Aug 25;112(5):1328–37. doi: 10.1093/ajcn/nqaa223 (PMC7657323; doi:10.1093/ajcn/nqaa223)
Supplement: nqaa223_Supplemental_File [file nqaa223_supplemental_file.pdf]

Schulze KJ et al., Newborn micronutrient status biomarkers in a cluster-randomized trial of antenatal multiple micronutrient versus iron-folic acid supplementation in rural Bangladesh

ONLINE SUPPLEMENTARY MATERIAL

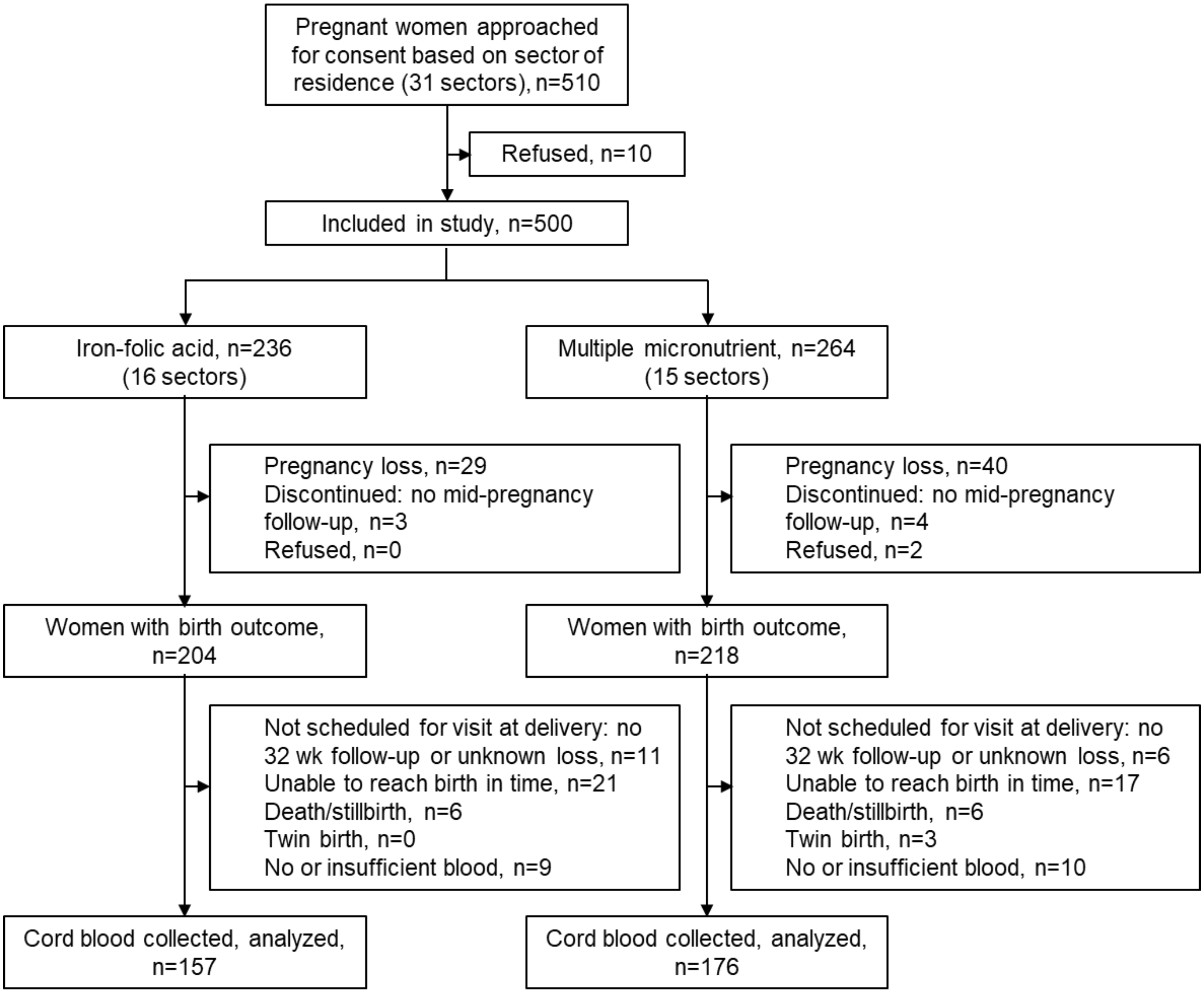

Supplementary Figure 1. Consolidated Standards of Reporting Trials diagram depicting source of cord blood samples for analysis of micronutrient biomarkers in Bangladeshi infants.

ONLINE SUPPLEMENTARY MATERIAL

Supplementary Table 1. Maternal micronutrient and inflammation biomarkers at 32 wk gestation by intervention status among the subset of mothers from the JiVitA-3 trial substudy in rural Bangladesh from whom cord blood was collected at the time of delivery

		IFA		MM		
Nutrient	Biomarker	N	Biomarker concentration	N	Biomarker concentration	P-value
Iron	Ferritin, µg/L	152	32 (15, 68)	165	33 (16, 65)	0.821
Folate	Total plasma folate, nmol/L	155	27 (13, 57)	174	25 (12, 52)	0.425
Vitamin B-12	Cobalamin, pmol/L	154	157 (107, 230)	174	168 (119, 236)	0.182
Vitamin A	Retinol, µmol/L	133	0.94±0.32	146	1.04±0.31	0.011
Vitamin D	25(OH)D, nmol/L	156	46 (35, 61)	174	52 (38, 71)	0.005
Vitamin E	α-Tocopherol, µmol/L	133	19.0±5.3	146	20.7±4.9	0.009
Zinc	Total plasma zinc, µmol/L	155	14.3 (11.7, 17.5)	176	15.2 (12.6, 18.4)	0.023
Iodine	Thyroglobulin, µg/L	149	35.3 (16.2, 77.2)	170	35.2 (18.6, 66.5)	0.475
	fT4, pmol/L	149	15.3±1.7	171	15.2±1.6	0.883
Inflammation	AGP, g/L	155	0.56 (0.38, 0.82)	174	0.54 (0.40, 0.74)	0.316

[1]Biomarker concentrations are geometric means (-1SD, +1SD) or mean±SD. Differences calculated from regression with biomarker or log10-biomarker as outcome, intervention as independent variable, and robust standard errors to account for cluster randomization. Conversion of biomarkers to other commonly used units can be done as follows: folate nmol/L x 0.4413 to ng/mL, B-12 pmol/L x 1.355 to pg/mL, retinol µmol/L x 28.65 to µg/dL, 25(OH)D pmol/L x 0.4006 to ng/mL, α-tocopherol µmol/L x 0.423 to µg/mL, zinc µmol/L x 6.534 to µg/dL, fT4 pmol/L x 0.0777 to ng/dL. AGP, α1-acid glycoprotein; fT4, free thyroxine; IFA, iron folic acid; MM, multiple micronutrients; 25(OH)D, 25-hydroxyvitamin D.

Supplementary Table 2. Distributions of newborn (cord blood plasma) micronutrient status and inflammation biomarkers among participants in the JiVitA-3 trial in rural Bangladesh[1]

Biomarker	N	Mean±SD	Geometric mean (-1SD, 1SD)	Selected percentiles						
				1st	10th	25th	50th	75th	90th	99th
Ferritin, µg/L	320	189±159	151 (76, 301)	44	70	110	158	223	311	900
Folate, nmol/L	332	65±32	58 (37, 93)	23	33	40	57	80	104	169
Cobalamin, pmol/L	331	345±193	298 (175, 509)	112	154	193	275	457	663	836
Retinol, µmol/L	282	0.64±0.23	0.60 (0.41, 0.88)	0.16	0.38	0.50	0.61	0.75	0.93	1.40
25(OH)D, nmol/L	333	44±16	41 (29, 59)	18	27	33	41	53	66	91
α-Tocopherol, µmol/L	282	6.0±2.0	5.6 (3.9, 8.1)	1.9	3.5	4.6	5.8	7.2	8.7	12.0
Zinc, µmol/L	331	15.1±3.1	14.8 (12.1, 18.0)	9.7	11.7	12.9	14.9	16.9	18.6	26.1
Thyroglobulin, µg/L	319	43.9±28.8	35.2 (17.4, 71.5)	5.9	15.5	22.3	39.0	57.5	80.5	143.0
fT4, pmol/L	320	15.2±1.6	15.1 (13.6, 16.9)	11.7	13.3	14.2	15.2	16.2	17.2	20.5
AGP, g/L	322	0.23±0.11	0.21 (0.13, 0.34)	0.05	0.11	0.15	0.21	0.29	0.37	0.60

[1] Conversion of biomarkers to other commonly used units can be done as follows: folate nmol/L x 0.4413 to ng/mL, cobalamin pmol/L x 1.355 to pg/mL, retinol µmol/L x 28.65 to µg/dL, 25(OH)D pmol/L x 0.4006 to ng/mL, α-tocopherol µmol/L x 0.423 to µg/mL, zinc µmol/L x 6.534 to µg/dL, fT4 pmol/L x 0.0777 to ng/dL. AGP, α1-acid glycoprotein; fT4, free thyroxine; IFA, iron folic acid; MM, multiple micronutrients; 25(OH)D, 25-hydroxyvitamin D.

Supplementary Table 3. Output for analysis to determine to effect of maternal micronutrient status in mediating the impact of MM versus IFA on improved newborn status among Bangladeshi infants[1]

Cord blood biomarker	n	Regression model 1[2]	Regression model 2[3]		Mediation analysis output[4]			
		Intervention on maternal status	Intervention on newborn status	Maternal status on newborn status	Average causal mediated effect	Direct effect	Total effect	Proportion of total effect mediated
		β (95%CI)	β (95%CI)	β (95%CI)	Mean (95%CI)	Mean (95%CI)	Mean (95%CI)	Mean (95%CI)
25(OH)D, nmol/L	326	0.374 (0.119, 0.629)*	0.022 (-0.007, 0.052)	0.101 (0.088, 0.113)**	0.038 (0.011, 0.064)*	0.022 (-0.008, 0.049)	0.060 (0.021, 0.098)*	0.624 (0.386, 1.797)*
Zinc, µmol/L	330	0.330 (0.048, 0.612)*	0.022 (0.002, 0.042)*	0.008 (0.000, 0.016)*	0.003 (-0.000, 0.007)	0.022 (0.001, 0.040)	0.024 (0.004, 0.043)	0.113 (0.062, 0.505)*

[1]Based on "medeff" command in Stata (references 17-19), to determine the extent to which the impact of the MM versus IFA supplement was mediated via the impact of the intervention on maternal micronutrient status, accounting for robust standard errors. IFA, iron folic acid; MM, multiple micronutrients; 25(OH)D, 25-hydroxyvitamin D.

[2]Regression model of intervention effect on maternal status equivalent to test shown in Supplementary Table 1. *P<0.05, **P<0.0001.

[3]Maternal and newborn biomarker data were $\log_{10}$-transformed for all biomarkers except retinol, α-tocopherol, and fT4; all maternal biomarker data was standardized. *P<0.05, **P<0.0001.

[4]"Average causal mediated effect" describes the strength of the effect of the MM intervention on cord blood biomarkers mediated through maternal biomarker status; "Direct effect" describes the direct impact of the MM versus IFA intervention on cord blood biomarkers; "Total effect" describes the combined direct and mediated effects of the MM versus IFA intervention on cord blood biomarkers; "Proportion of total effect mediated" is estimated from the average causal mediated effect relative to the total effect. Values where 95%CI do not overlap zero are denoted with *, although no P-value is established for these estimates.

Supplementary Table 4. Additional regression models with variables with significant associations with newborn micronutrient biomarker concentrations from the JiVitA-3 trial in rural Bangladesh

Newborn Biomarkers (outcomes)	Model	Independent Variables[1]	β-coefficient (95%CI)	*P*-value	n	R^2	Prob>F
Retinol[3], µmol/L	1	Intervention (MM vs IFA)	0.032 (-0.029, 0.092)	0.295	277	0.1019	0.0001
		Maternal biomarker	0.016 (-0.007, 0.039)	0.169			
		Weight-for-age Z-score at birth	0.070 (0.041, 0.099)	<0.0001			
	2	Intervention (MM vs IFA)	0.029 (-0.033, 0.092)	0.346	276	0.0377	0.0179
		Maternal biomarker	0.019 (-0.006, 0.045)	0.133			
		Length-for-age Z-score at birth	0.034 (0.007, 0.062)	0.016			
	3	Intervention (MM vs IFA)	0.010 (-0.050, 0.069)	0.738	248	0.0767	0.0053
		Maternal biomarker	0.012 (-0.013, 0.037)	0.343			
		Weight-for-length Z-score at birth	0.059 (0.027, 0.091)	0.001			
	4	Intervention (MM vs IFA)	0.016 (-0.047, 0.079)	0.604	278	0.0335	0.0237
		Maternal biomarker	0.020 (-0.005, 0.045)	0.119			
		Cord blood AGP, $\log_{10}$g/L	0.163 (0.011, 0.315)	0.036			
α-Tocopherol, µmol/L	1	Intervention (MM vs IFA)	0.249 (-0.351, 0.837)	0.410	278	0.0906	0.0005
		Maternal biomarker	0.470 (0.143, 0.796)	0.006			
		Cord blood AGP, $\log_{10}$g/L	1.55 (0.338, 2.762)	0.014			
fT4, pmol/L	1	Intervention (MM vs IFA)	-0.083 (-0.470, 0.304)	0.664	227	0.0798	0.0197
		Maternal biomarker	0.099 (-0.140, 0.338)	0.403			
		Maternal biomarker (early pregnancy)	0.121 (0.037, 0.205)	0.006			

[1]Maternal biomarkers are from 32 weeks gestation unless otherwise noted and distributions were standardized.

[2]Newborn weight (kg) and length (cm) were also positively—and newborn underweight (weight-for-age Z-score <-2), stunting (length-for-age Z-score <-2), and wasting (weight-for-length Z-score<-2) were also negatively—associated with cord retinol concentrations, *P*<0.05.

AGP, α1-acid glycoprotein; fT4, free thyroxine; IFA, iron folic acid; MM, multiple micronutrients; 25(OH)D, 25-hydroxyvitamin D.
